# Tracking Evidences of Dandelion for the Treatment of Cancer: From Chemical Composition, Bioactivity, Signaling Pathways in Cancer Cells to Perspective Study

**DOI:** 10.3390/nu17233769

**Published:** 2025-11-30

**Authors:** Anqi Wang, Wugui Xiong, Cui Cheng, Liang Zou, Bei Niu, Ying Liu

**Affiliations:** 1 School of Food and Bioengineering, Institute for Advanced Study, Chengdu University, Chengdu 610106, China; wanganqi@cdu.edu.cn (A.W.);; 2 School of Preclinical Medicine, Chengdu University, Chengdu 610106, China; 3 Antibiotics Research and Re-Evaluation Key Laboratory of Sichuan Province, Sichuan Industrial Institute of Antibiotics, Chengdu University, Chengdu 610052, China

**Keywords:** dandelion, *Taraxacum*, anticancer, signaling pathways, cell apoptosis, cell cycle, cellular metabolism

## Abstract

Cancer has become the second leading cause of death globally and is a big threat to human health. The development of new anticancer drugs and the elucidation of the signaling pathways of bioactive compounds are still effective strategies to address the current challenges in the clinical treatment of cancer. Dietary compounds are composed of a variety of effective ingredients, which have become an important source for the development of novel candidates for the treatment of cancer. These bioactive ingredients often carry the characteristics of low side effects, multi-target, and economic savings and hence attract more and more scholars’ research interests in them. Dandelion, one of the important medicinal and edible plants, is effective in anticancer, inhibition of bacterial growth, hypoglycemic, and anti-inflammation, as well as antioxidant. Growing evidences from modern pharmacological research demonstrate the notable anticancer effects of dandelion. Bioactive components from dandelion are effective in inhibiting the occurrence and progression of various cancers, such as breast, lung, and liver cancers. Hence, the chemical composition, anticancer activities, and signaling pathways in cancer cells treated with bioactive components from dandelion are summarized in this review. We aim to provide more pharmacological evidence and scientific references for further research and development of dandelion for cancer treatment. Meanwhile, we anticipate that some novel candidates with high efficacy and low toxicity for anticancer might be developed from dandelion in future research on this plant.

## 1. Introduction

Cancer, one of the common and complex diseases, has been considered the second leading cause of death globally and a big threat to human health [1]. According to the recent research report, there were 20 million new cancer cases and 9.7 million deaths worldwide in 2022, which causes a huge social burden for the management of this disease [2]. The morbidity and mortality of cancer in China rank first in the world [2]. The conventional methods for clinical treatment of cancer mainly include surgical treatment, chemotherapy, and radiotherapy. The surgical treatment is often not suitable for patients in the middle and late stages of cancer. Although radiotherapy and chemotherapy are the standard methods for the treatment of cancer, the prognosis and efficacy are unsatisfactory, which are accompanied by serious side effects and tumor resistance [3]. Therefore, more effort should be exerted to develop a strategy with safety, efficacy, low toxicity and easily available for the treatment of cancer.

Natural products derived from medicinal and edible plants have always been an important source for research and development of food supplements or drugs for the treatment of cancer, as they have been widely used alone or in combination with other drugs in ancient times [4]. Compared with chemical drugs, bioactive natural products have more obvious advantages and broad application prospects for the prevention and treatment of cancer. On the one hand, natural products are often composed of a variety of active ingredients, which may exert overlapping or synergistic effects for anti-cancer through multiple targets and pathways [5]. On the other hand, natural products have advantages, such as few side effects, extensive sources, and easy access, resulting in economical savings [6]. Therefore, the research interests in developing natural products from medicinal and edible plants for the treatment or prevention of cancer have increased rapidly in recent years.

Dandelions are the plants of *Taraxacum monogolicum* Hand.-Mazz., *Taraxacum borealisinense* Kitam, and other species from the *Taraxacum* genus. The growing number of research evidences presents the novel and potential effect of dandelion in treating or preventing cancers, including breast cancer, gastric cancer, colorectal cancer, and other malignant tumors [7,8,9]. Additionally, pharmacological studies point out that the extract and bioactive components of dandelion show significant anti-inflammatory, anti-oxidation, and other activities [10]. Through searching for the data of preparations and health products containing dandelion, it is found that there are currently two preparations containing dandelion sold in China, which are used for the treatment of liver or breast-related diseases. In our country, dandelion and its extracts are also widely involved in 82 types of food supplements, which are used to improve immunity, protect the liver or gastric mucosa, etc. Meanwhile, the extracts of dandelion are also used as bioactive ingredients and added to some cosmetics. All of these evidences indicate the great research value of dandelion in the development of drugs or health products.

To date, although a large number of studies have presented the inhibitory effects of dandelion or its extracts on breast, lung, liver and other cancers, the underlying molecular mechanisms are still unclear, which greatly limits its development and clinical application. Therefore, in this paper, the chemical composition of dandelion, and the anti-tumor mechanisms are comprehensively reviewed, which aims to provide a pharmacological or nutritional reference for the development of dandelion in future.

## 2. The Nutritional and Development Value of Dandelion

### 2.1. Development of Fresh Food and Edible Products

Dandelion is rich in proteins (2–3 g/100 g fresh plant), carbohydrates (3–5 g/100 g fresh plant), and amino acids, and contains more than 60 kinds of trace elements, such as calcium, iron, phosphorus, zinc, and manganese. The content of vitamin A in dandelion is about 14,000 IU/100 g fresh plant. With deep research and development of medicinal and edible plants, the nutritional and health care values of dandelion are closely integrated with food development, and many novel functional supplementary foods have been developed, which are enriching our daily food structure. Dandelion, together with millet and soybean, is used as a raw material to develop dandelion miscellaneous grains biscuits, and the health care effect of the biscuits is expanded by adding dandelion [11]. The ultrasonic-assisted extraction of dandelion dry leaves is also used as a raw material for the preparation of a beverage, which has a soft taste and a unique aroma after the deployment of citrus and dandelion [12]. In addition, the whole plant of dandelion can be processed into powder, which can be taken separately, and can also be used as auxiliary materials to make dandelion-flavored steamed bread, noodles, and so on. In recent years, the research interests in extracting and utilizing the active ingredients of dandelion have gradually increased. For example, a natural edible yellow pigment found in dandelion, which is green and safe and can be widely used in food production [13]. The root of the dandelion is rich in polysaccharides, which are suitable for being extracted and used to develop functional beverages with unique tastes [14].

### 2.2. Development of Dandelion-Related Health Products

The wide range of investigations demonstrates that the extracts of dandelion are effective in antibacterial, anti-inflammatory, anti-oxidation, anti-tumor, immune regulation, and other effects [15]. The research interests of scholars are not only limited to the clinical application and edible value of dandelion but also focus on exploring and developing dandelion into various health care products. Dandelion is used as one of the main materials to develop a rash-removing solution, which can be used for the treatment of eczema dermatitis symptoms and can be used as a physiotherapy solution for a long time without any side effects [16]. Research also points out that dandelions are effective in freckle whitening and skin clearing, thus they can be used as one of the materials to develop a dandelion acne-removing facial mask, which aims to improve skin darkness, roughness, relaxation, and other problems with a natural affinity and no irritation to the skin [17]. The extract of dandelion can also be made into dandelion dairy products, health toothpaste, soap, and so on [18].

### 2.3. Development of Dandelion Characteristic Tea Products

Using different processing methods, the leaves of dandelion can be processed into green and black tea. Research demonstrated that more unique polyphenols and flavonoids in dandelion were preserved in black tea [19]. The leaves and roots of dandelion are usually made into tea. However, the dandelion root is rich in dandelion alcohol, dandelion sorbitol, organic acid and polysaccharides. Consequently, dandelion root tea has a stronger sweet taste, is more resistant to brewing, and has a stronger hepatoprotective effect compared to dandelion leaf tea [20].

## 3. Chemical Composition

Different extraction methods are often used for extracting different kinds of components from this plant. Ultrasonic extraction, microwave-assisted extraction, and hot reflux extraction are usually used for the extraction of terpenoids. Enzymatic extraction, ultrasonic extraction, and hot reflux extraction are often used for the extraction of polysaccharides. Ultrasonic extraction and microwave-assisted extraction are used for the extraction of flavonoids. To date, reports have mainly focused on the extraction rates of flavonoids and polysaccharides from this plant [21,22].

Studies demonstrate that the levels of chlorogenic acid, caffeic acid, polysaccharides, flavonoids, saponins, and other components in dandelion are higher during the vigorous growth period than during the other periods [23]. The contents of components in dandelion are determined by harvesting time and by different parts of the plant [23]. It is reported that the flowers have the most abundant types of chemical components, while the leaves account for the highest content of chicoric acid [24].

To date, various chemical components have been isolated from dandelion, including triterpenoids, polysaccharides, flavonoids, phenolic acids, sesquiterpenoids, coumarins, fatty acids, organic acids, and pigments. In addition, dandelion is also rich in vitamins (A, B, C, D, and E), amino acids, carbohydrates, choline, inositol, lecithin, minerals, and trace elements (calcium, sodium, magnesium, iron, silicon, copper, phosphorus, zinc, manganese, and potassium) [10]. The main chemical components from this plant are summarized in Table 1 and the following parts.

### 3.1. Flavonoids

Flavonoids are one of the most abundant components in dandelion and the main secondary metabolites of this plant. The flavonoids in dandelion all belong to 2-phenylchromone. Ten flavonoids, including luteolin, quercetin, luteolin-7-O-β-D-glucoside, luteolin-4-O-β-D-glucoside, and luteolin-3-O-β-D-glucoside, are identified [25]. In addition, research reported that dandelion mainly contains flavonoids, such as luteolin, quercetin, luteolin-7-O-β-D-glucoside, luteolin-4-O-β-D-glucoside, luteolin-3-O-β-D-glucoside, luteolin-7-O-β-D-rutinoside, luteolin-7-O-β-D-gentiopyranoside, quercetin-7-O-β-D-glucoside, isorhamnetin-3-O-β-D-glucoside, and rhamnetin-3,7-O-β-D-diglucoside [26]. The flavonoids from dandelion are widely reported to be effective in antioxidation and inhibition of porcine pancreatic α-amylase [29,33].

### 3.2. Terpenoids

The terpenoids in dandelion are mainly composed of triterpenoids and sesquiterpenoids, and these components are considered to be related to its anti-inflammation and anticancer effects. The terpenoids in dandelion are mainly pentacyclic triterpenoids. Katrin et al. isolated and identified taraxastrol, pseudotaraxastrol, and their hexadecyl derivatives (taraxastrol-2,3-diol and columbianadin-2,3-diol) from dandelion roots [27]. Warashina et al. isolated eight new triterpenoids from the methanol extract of dandelion, including 21-peroxyhydroxy-taraxastrol acetate and 30-peroxyhydroxy-pseudotaraxastrol acetate, etc. [30]. In addition, taraxastrol and oleanolic acid were also identified in dandelion. Triterpene biosynthesis occurs through the action of oxidos-qualene cyclase (OSC), which generates various types of triterpenes from 2,3-oxidosqualene after the rearrangement of the triterpene skeleton [34]. Transcriptome analysis of the putative OSC genes in *Taraxacum coreanum* demonstrates that TcOSC1 produced several triterpenes, including taraxasterol; ψ-taraxasterol; α-, β- and δ- amyrin; and dammarenediol-II. TcOSC2 catalyzed the production of bauerenol and another unknown triterpene; TcOSC3 catalyzed the production of β-amyrin [34]. TcOSC4 catalyzed the production of taraxerol. These enzymes are novel triterpene synthases that participate in the production of taraxasterol, bauerenol, and taraxerol [34].

Sesquiterpenoids are the main components in dandelion and are responsible for the bitter taste of this plant. Shi et al. isolated and identified four sesquiterpenoids from dandelion, including taraxastatin, isodonsesquitin A, taraxastatin B, and sesquiterpene lactone [28]. In another report, artecalin, arsanin, and desacetylmatricarin were isolated from this plant for the first time [29]. Dandelion also contains dandelion acid β-D-glucopyranoside, 11β,13-dihydro-lactucarium, and dandelion lactone β-D-glucoside [27].

### 3.3. Phenolic Acids

Phenolic acids, the most important secondary metabolites, are also abundant in dandelion. Currently, the phenolic acids isolated and identified from dandelion include p-hydroxybenzoic acid, phenylacetic acid, protocatechuic acid, p-coumaric acid, caffeic acid, ferulic acid, syringic acid, vanillic acid, chlorogenic acid, 3,5-O-dicaffeoylquinic acid, 3,4-O-dicaffeoylquinic acid, 4,5-O-dicaffeoylquinic acid, 3,5-dihydroxybenzoic acid, gallic acid, 3,4-dihydroxybenzoic acid, *p*-hydroxyphenylacetic acid, methyl *p*-hydroxyphenylacetate, ethyl p-hydroxyphenylacetate, ethyl caffeate, methyl caffeate, tartaric acid, cichoric acid, monocaffeoyl tartaric acid, 1-hydroxymethyl-5-hydroxy-benzene-2-O-β-D-glucopyranoside, coumaric acid, methyl gallate, 4-O-caffeoylquinic acid, and vanillic acid. [26,28,31,32]. The phenolic acids from dandelion are often correlated with its antioxidation effect [35].

### 3.4. Polysaccharides

Polysaccharides are kinds of high-molecular carbohydrates formed by condensation, dehydration and polymerization of more than 10 monosaccharides, which are ubiquitously found in different plants. As important components of plants, polysaccharides present various biological activities, such as anti-tumor, anti-oxidation, and immune regulation [15]. Currently, research on the chemical structure of dandelion polysaccharides mainly focuses on the primary structure, including the composition and ratio of monosaccharides, the linkage mode of monosaccharides, and molecular weight and glycosidic bond types. Polysaccharides from dandelion are composed of a variety of monosaccharides, including D-rhamnose, glucose, D-galactose, D-xylose, and D-arabinose. Wang et al. isolated a new polysaccharide (DLP-I) with a molecular weight of 87,000 g/mol from the leaf of dandelion. Structural characterization showed that DLP-I was composed of five monosaccharides, including galactose, rhamnose, arabinose, glucose, and mannose, with a molar ratio of 1.14:1.00:1.05:4.76:1.52. 1D and 2D NMR spectra confirmed the sugar chain structure of DLP-I was → 4) -α-D-Galp- (1 →, → 4) -β-D-Manp- (1 →, → 4) -α-D-Glcp- (1 →, → 2,4) -α-L-Rhap- (1 →, and α-L-Araf- (1 → with branching at O-2 and O-4 of → 2,4) -α-L-Rhap- (1 → [21]. Cai et al. identified two novel polysaccharides (DRP-2b, DRP-3a) from the root of dandelion [22]. Structural analysis showed that the molecular weight of DRP-2b was 31.8 kDa, which was composed of rhamnose, glucuronic acid, glucose, galactose, and arabinose, and the main chain was (1 → 5) -α-D-Ara. The molecular weight of DRP-3a was 6.72 kDa, which was composed of rhamnose, glucose, galactose, and arabinose. The main chain was (1 → 6)-α-D-Glc [22]. Actually, due to the complexity of the polysaccharides, the chemical information of other polysaccharides from dandelion still needs to be further disclosed.

## 4. Signaling Pathways in Cancer Cells by Bioactive Components from Dandelion

**Figure 1:**
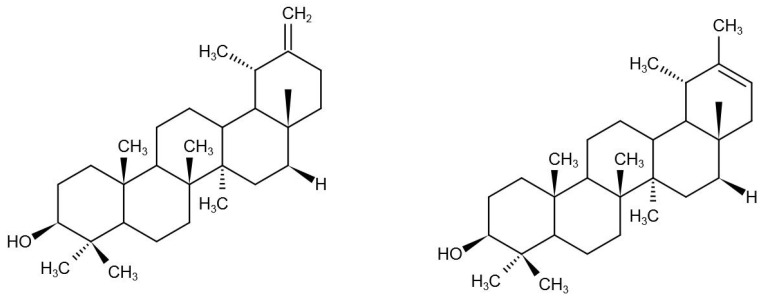
The chemical structure of taraxasterol (**left**) and ψ-taraxasterol (**right**) isolated and identified from dandelion.

It is reported that extracts and some bioactive ingredients from dandelion are effective in the treatment of a variety of tumor cells, including breast cancer, lung cancer, colorectal cancer, pancreatic cancer, liver cancer, gastric cancer, and bladder cancer. Different signaling pathways are involved in the process of regulating cancer cells in vitro and vivo, and the signaling pathways in cancer cells by bioactive components from dandelion are reviewed in the following section. According to current reports, extracts, polysaccharides, ψ-taraxasterol, and taraxasterol from dandelion are mainly responsible for its anticancer activities. The anticancer capacity of other kinds of components from this plant is rarely reported. The chemical structures of taraxasterol and ψ-taraxasterol are shown in Figure 1.

### 4.1. Cell Cycle Arrest and Anti-Proliferation in Cancer Cells

The cell cycle regulators often play a crucial role in cell division and proliferation. The abnormal expression of these regulators is often accompanied by excessive division and proliferation of tumor cells, resulting in the uncontrolled spread and metastasis of cancer cells. Therefore, inhibition of the abnormal expression of cell cycle regulators is a key to suppressing the proliferation of cancer cells, which is also considered an important strategy and the primary goal of screening and developing anti-tumor drugs [36].

ψ-taraxasterol (1 µM) blocks the cell cycle of gastric cancer cells at the G0/G1 phase through downregulating the expression of cyclin D1 and proliferating cell nuclear antigen (PCNA) and upregulating the expression of p21, thereby inhibiting the proliferation of HGC-27 and NCI-N87 cells [37]. The ex vivo experiment demonstrates that taraxasterol is effective in inhibiting the proliferation of human hepatocellular carcinoma SK-Hep1 and HepG2 cells by upregulating the expression of histidine triad nucleotide-binding protein 1 (Hint1) and downregulating the expression of cyclin D1, which arrests the cell cycle at the G0/G1 phase. The IC_50_ values for taraxasterol in SK-Hep1 and HepG2 cells are 17.0 and 9.9 µM, respectively [38]. Taraxasterol downregulates the expression of Ki-67 to inhibit the tumor growth in hepatocellular carcinoma-bearing mice in a dose-dependent manner (5.0–7.5 mg/kg B.W.) [39]. It is also reported that the growth of gastric cancer cells in mice is significantly inhibited by treatment with 25 µg/mL of taraxasterol for 16 days, and a mechanism study demonstrates that taraxasterol inhibited the growth of gastric cancer cells in mice by inhibition of EGFR/AKT1 signaling [40]. In addition, polysaccharides from dandelion significantly inhibit the proliferation of MCF-7 cells by upregulating the expression of the cell cycle regulatory factor and tumor suppressor gene *p53* in a dose-dependent manner (50–400 µg/mL) [41].

### 4.2. Induction of Cell Apoptosis in Cancer Cells

Two major pathways participate in the induction of cell apoptosis, including the endogenous pathway involving mitochondrial stress and the exogenous pathway mediated by death receptors. The mitochondrial pathway is primarily activated by apoptosis-stimulating factors, such as hypoxia, infection, increased intracellular calcium ion concentration, and oxidative stress. These apoptosis-stimulating factors activate the expression of Bcl-2-associated X protein (Bax) and Bcl-2 antagonist/killer (Bak) in the B-cell lymphoma-2 (Bcl-2) protein family. These proteins bind to the outer mitochondrial membrane to form Bax-Bak pores, leading to the increased permeability of the outer mitochondrial membrane and mediating the release of cytochrome c (Cyt-c). Cyt-c that is released into the cytoplasm combines with apoptotic protease activating factor-1 (Apaf-1) and cystein asparate protease-9 (Caspase-9) to form an apoptotic body, which then activates Caspase-3 to trigger the Caspase cascade reaction and induce cell apoptosis. In death receptor-mediated cell apoptosis, the signal transduction cascade is activated through the binding of death signals to the death receptor ligands (such as Fas/FasL and tumor necrosis factor). Upon receiving extracellular death signals, Caspase-8 is activated and then triggers the activation of downstream Caspases and induces apoptosis [42].

The mechanisms of inducing cell apoptosis in cancer cells by dandelion are mainly involved in mitochondrial stress. The extract of dandelion at a concentration of 100 mg/mL can significantly induce cell apoptosis in pediatric tumor cell lines SH-SY5Y and Kelly by disrupting mitochondrial integrity, with apoptosis rates of 66.6% and 66.0%, respectively [43]. The extract from dandelion root induces cell apoptosis by disrupting mitochondrial membrane potential, activating Caspase-9 and Caspase-3, and downregulating the expression of Bcl-2 in human breast cancer MDA-MB-231 [44]. In addition, the extract from dandelion root inhibits the expression of Bcl-2 and the activation of the phosphoinositide 3-kinase (PI3K)/protein kinase B (Akt) signaling pathway in a dose-dependent manner to induce cell apoptosis in human tongue cancer Tca-8113 cells [45].

Apart from investigation on the anticancer activities of the extract of dandelion, the effects of inducing cell apoptosis by ψ-taraxasterol are also reported by researchers. ψ-taraxasterol upregulates the expression of Bax and downregulates the expression of Bcl-2 in a dose-dependent manner, which leads to the release of Cyt-c and activation of Caspase-9, thereby inducing apoptosis in human cervical cancer HeLa cells and human gastric cancer HGC-27 and NCI-N87 cells. The mechanisms of its action may be related to the inhibition of the PI3K/Akt signaling pathway in cells [37,46].

### 4.3. Inhibition of Migration and Invasion of Cancer Cells

Multiple links and factors participate in the process of migration and invasion for cancer cells [47]. It is confirmed that the epithelial-mesenchymal transition (EMT) for cells plays a significant role in tumor migration and invasion. In the EMT process, the adhesion of cancer cells is reduced, and the characteristics of epithelial cells are diminished, with enhanced characteristics of mesenchymal cells. It is specifically manifested by the increased expression of N-cadherin and decreased expression of E-cadherin in cancer cells [48].

Components from dandelion presented promising effects in regulating the progression of EMT for cancer cells. The migration rates of human esophageal squamous cell carcinoma KYSE30 and TE-1 cells treated with 0.5 µg/mL of flavonoids from the ethanol extract of dandelion are significantly reduced to 19.33% and 18.32%, respectively, and the invasion rates of the cells are decreased to 31.12% and 14.54%, respectively [49]. The downregulation of zinc finger transcription factor-1, zonula occludens-1, and N-cadherin, as well as the upregulation of the expression of E-cadherin, is related to its anticancer action [49].

The expression of matrix metalloproteinases (MMPs) in cancer cells is often significantly increased in the progression of EMT. Consequently, inhibition of the expression of MMPs is also crucial for suppressing the invasion and metastasis of cancer cells. Study demonstrates that the rate of cell migration and invasion of BGC823 cells treated with 2.5 mg/mL of the extract from dandelion is reduced to 64.64% and 68.80%, respectively [50]. It is deduced that the decreased expression of *MMP-2* mRNA in BGC823 cells is responsible for the treatment with the extract from dandelion [50]. In addition, evidence shows that taraxasterol inhibits the migration and invasion of human thyroid papillary carcinoma TPC-1 and BCPAP cells by regulating Wnt/β-catenin signaling and suppressing the expression of MMP-2 and MMP-9 [51].

Growing evidences point out that the expression of some long non-coding RNA (LncRNA) also plays a vital role in the regulation of the migration and invasion of cancer cells [52,53]. The extract from dandelion root (3 mg/mL) is reported to inhibit the migration and invasion of human gastric cancer SGC7901 and BGC823 cells by downregulation of the expression of LncRNA CCAT-1 [8]. Similarly, ψ-taraxasterol increases the adhesion between human bladder cancer EJ cells by inhibiting the expression of EMT-related proteins mediated by LncRNA α/β hydrolase domain 11 antisense RNA1. Compared with the control group, ψ-taraxasterol at a concentration of 100 µM can reduce the rates of cell invasion and migration by 68.23% and 51.05%, respectively [54].

The migration and invasion of cancer cells always depend on complex interactions between different signaling molecules. The extract of dandelion blocks the PI3K/Akt and Ras/Raf/Erk signaling pathways in esophageal squamous cell carcinoma KYSE 450 and NEC cells (with IC50 values of 11.34 and 6.97 mg/mL, respectively), and this signaling plays a key role in controlling the migration and invasion of cells [55]. Deng et al. report that dandelion extract exerts inhibitory effects on STAT3 and PD-L1 in TNBC cells under a tumor-activated macrophage microenvironment. Furthermore, dandelion extract remarkably promotes the expression of M1-like marker TNF-α, IL-8, and iNOS but reduces the M2-like marker IL-10, CD206, Arginase-1, and TGF-β in M2 macrophages [56].

### 4.4. Induction of Autophagy in Cancer Cells

Autophagy, a process of degrading and recycling proteins and organelles, plays a crucial role in maintaining the intracellular homeostasis [57]. In cancer cells, autophagy often plays a dual role in regulating their activities. On the one hand, autophagy in cancer cells provides nutrients to sustain their survival. On the other hand, excessive induction of autophagy will lead to autophagic death of cancer cells, thus providing a new approach for the treatment of cancer [58].

Research demonstrates that the bioactive components from dandelion exert their anticancer effects by induction of excessive autophagy in cancer cells. ψ-taraxasterol induces excessive autophagy in MCF-7 cells by targeting and inhibiting the mTOR/ eukaryotic translation initiation factor 4E binding protein 1 (4EBP1) pathway. In this experiment, the mTOR/4EBP1 pathway is found to be significantly inhibited, and the expression of the autophagy-related protein yeast Atg6 homolog (Beclin1) is upregulated, which leads to the conversion of the microtubule-associated protein 1 light chain 3-I (LC3-I) to LC3-II [59]. In addition, taraxasterol (50 µg/mL) significantly induces excessive autophagy to inhibit the proliferation of human colon cancer HCT116 and SW480 cells, in which taraxasterol promotes the degradation of pro-cancer gene ring finger protein 31 (RNF31) [60]. It is also found that RNF31 often interacts with p53 and promotes p53 ubiquitination and degradation. The PUB domain of RNF31 is the key structure in the induction of p53 ubiquitination [60]. The induction of autophagy in cancer cells by ψ-taraxasterol and taraxasterol has been investigated in cells with no in vivo studies, and further studies should be performed to investigate the activities in animals and selectivity between normal cells and cancer cells.

### 4.5. Regulation of the Metabolism of Cancer Cells

Metabolic reprogramming is one of the most important hallmarks of malignant tumors, which is a crucial strategy for their survival and proliferation, involving tissue- and condition-dependent remodeling of certain metabolic pathways [61,62]. Even under conditions of abundant oxygen, cancer cells tend to convert glucose into lactic acid, a process known as the Warburg effect, which facilitates the proliferation of various cancers [63,64]. Therefore, inducing glycolysis shift in cancer cells is beneficial for inhibiting the occurrence and development of tumors. The bioactive components of dandelion exert their anti-tumor effects by regulating the metabolism of glucose in cancer cells through multiple pathways. Glycolysis in human lung cancer H1299 and A549 cells is inhibited by the treatment with ψ-taraxasterol through decreasing the activity of hexokinase [65]. Taraxasterol significantly reduces the level of glyceraldehyde-3-phosphate dehydrogenase in aerobic glycolysis, which is beneficial for suppressing cell proliferation and boosting cell apoptosis in HGC-27 cells [66].

Unlike normal cells, cancer cells always depend on an increased amount of iron for their survival and proliferation, and macrophages may transfer iron to cancer cells to promote carcinogenesis [67]. Thus, intervention of the iron metabolism in cancer cells is also a potential strategy for the treatment of cancer. Polysaccharides from dandelion (200 mg/L) is reported to be effective in anticancer by regulating iron metabolism in cancer cells. Decreased expression of iron metabolism-related genes, including hepcidin, ferroportin, and iron overload, in human hepatocellular carcinoma Huh7 and HepG2 cells is responsible for its antiproliferation of cancer cells [68]. Meanwhile, polysaccharides from dandelion (200 mg/kg B.W.) significantly inhibit iron deposition in tumor tissues of mice bearing hepatocellular carcinoma Hepa1-6 and H22 cells [68].

Additionally, abnormal lipid metabolism is another important characteristic of malignant tumors, since a large amount of lipid is required for cancer cells to satisfy the formation of various organelles and some specific needs [69]. In cancer cells, phosphatidylcholines (PC) are synthesized via the Kennedy pathway, in which choline is converted into choline phosphate by choline kinase and then into cytidine diphosphate choline (CDPC). In the final step, the PC is synthesized by condensation of CDPC and diacylglycerol. The report demonstrates that the dandelion extract exerts anti-tumor effects by inhibiting the synthesis of PC and glycerophospholipid metabolism pathways [70].

### 4.6. Anti-Angiogenesis in Cancer Cells

Angiogenesis is also a crucial process for tumor growth and metastasis, and an increased number of blood vessels is beneficial for providing sufficient nutrients for cancer cells and accelerating their growth. Therefore, inhibition of angiogenesis in cancer cells is considered an effective way to suppress tumor growth and metastasis [71]. The anticancer effects of dandelion polysaccharides for hepatocellular carcinoma are just related to the downregulation of the expression of hypoxia-inducible factor-1α and vascular endothelial growth factor (VEGF) in cancer cells [72]. The main signaling pathways responsible for the bioactive components from dandelion for the treatment of cancer are summarized in Table 2.

### 4.7. Other Pathways

**Figure 2:**
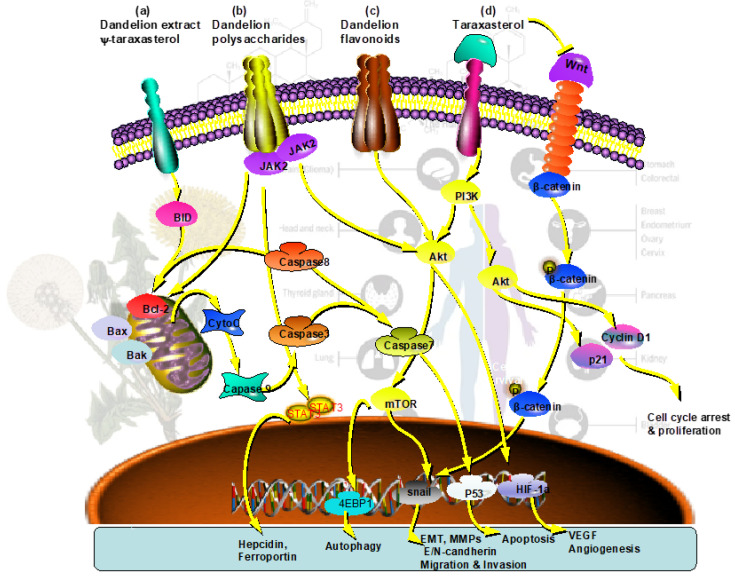
The diagram of the main signaling pathways responsible for the bioactive components from dandelion for the treatment of cancer; (**a**) the dandelion extract and ψ-taraxasterol involved in the Bcl-2/caspase related cell apoptosis signaling; (**b**) the dandelion polysaccharides involved in iron metabolism, angiogenesis, and cell apoptosis; (**c**) the dandelion flavonoids involved in E/N-candherin for regulation of cell migration and invasion; (**d**) taraxasterol involved in cell cycle arrest and antiproliferation, induction of apoptosis, inhibition of invasion and metastasis, and autophagy in cancer cells.

Many studies have shown that bioactive components from dandelion can enhance the therapeutic effect of chemical drugs and reduce their damage to the human body [73,74]. The dandelion root extract combined with paclitaxel and mitoxantrone presents a synergistic pro-apoptotic effect on cancer cells. The sensitivity of human prostate cancer DU-145 cells to paclitaxel and mitoxantrone is significantly enhanced, and the tumor volume in mice with human prostate cancer xenografts is significantly decreased by combination with the dandelion root extract [75]. The sensitivity of cyclophosphamide for Lewis lung cancer mice is also significantly increased by the combination treatment with flavonoids from dandelion [76]. In another report, dandelion seed extract can enhance the sensitivity of cisplatin for human esophageal squamous cell carcinoma [77]. It is also reported that the dandelion aqueous extract can not only increase the sensitivity of tumor cells to doxorubicin but also can reduce doxorubicin-induced cardiotoxicity [74]. The main signaling pathways responsible for the bioactive components from dandelion for the treatment of cancer are illustrated in Figure 2.

## 5. Perspectives

The high incidence and serious mortality of cancer pose a serious threat to human health and life. Although the common radio and chemotherapy for malignant tumors can inhibit tumor progression, many serious side effects and multi-drug resistance caused by the treatment are inevitable. Natural products have advantages in abundant chemical structures, extensive anti-tumor activities, and easy access at low cost. And they are considered a great treasure for the development of drugs or health products for the treatment or prevention of cancer.

To date, research on the anti-tumor effects of dandelion mainly focuses on dandelion root extracts, polysaccharides, ψ-taraxasterol, and taraxasterol, but less attention is paid to the anti-tumor effects and mechanisms of other components of dandelion. Moreover, most studies on the anti-tumor effects of active components from dandelion are just concentrated on basic pharmacological mechanisms, and research on signaling mechanisms tends to be too monotonous. It is suggested that more modern pharmacological techniques and methodologies be used to enrich the study on the mechanisms of bioactive components from dandelion for their anticancer activities. For example, one study comprehensively demonstrates the multi-target mechanism for dandelion in the treatment of triple-negative breast cancer using network pharmacology, molecular pharmacology, and a metabolomics approach [78]. This kind of research paradigm will provide new insights for researchers to identify, confirm, and optimize relationships between bioactive components in dandelion and targets that contribute to their anticancer effects. In addition, most of the current studies are primarily performed on in vitro cancer cells, and the real anticancer capacity of dandelion should also be investigated on animals.

In future research, extensive in vivo animal models are quite necessary and indispensable for further investigating the signaling pathways and bioactive components in dandelion. To ensure the further development and utilization of bioactive ingredients in dandelion for the treatment of cancer, it is also indispensable to investigate their pharmacokinetic behaviors in vivo, and such research is still unreported till today. Although current research demonstrates the anticancer effects of triterpenoids and other components in dandelion, it is found that the effective concentrations for most of them are too high and limit their development value, which may be related to their weak activity or lack of specificity in therapeutic targets. In future research, these potential bioactive ingredients may be used as skeletons to develop hit compounds through chemical modification for the treatment of cancer with stronger activity and a specific target.

## 6. Conclusions

The anticancer effects of compounds in dandelion have been extensively studied. Results demonstrate that these compounds exhibit no toxicity toward normal cells and mice, and present certain advantages and potential applications in the research and development of anticancer drugs or adjuvant therapy. The potential signaling pathways for bioactive components in dandelion in cancer cells are reviewed in this article. Briefly, first of all, bioactive components in dandelion inhibit the cell cycle progression and cell proliferation of cancer cells by suppressing the cyclin D1/p21 pathway and promoting p53 expression. Secondly, bioactive components in dandelion induce tumor cell apoptosis through the mitochondrial Bcl-2/caspase pathway. Additionally, bioactive components in dandelion inhibit tumor cell migration and invasion by suppressing the EMT progression, MMPs expression, and blocking the PI3K/Akt pathway. Additionally, regulation of cell metabolism, induction of autophagy, and inhibition of angiogenesis are also involved in the signaling pathways for the treatment of cancer by bioactive components in dandelion.

## Figures and Tables

**Table 1:** Brief information on representative compounds isolated and identified from dandelion.

NO	Types of Components	Representative Compounds	Reference
1	Flavonoids	luteolin	[25]
		quercetin	[25]
		luteolin-7-O-β-D-glucoside	[25]
		luteolin-4-O-β-D-glucoside	[25]
		luteolin-3-O-β-D-glucoside	[25]
		quercetin-7-O-β-D-glucoside	[26]
		isorhamnetin-3-O-β-D-glucoside	[26]
		rhamnetin-3,7-O-β-D-diglucoside	[26]
2	Terpenoids	taraxastrol	[27]
		pseudotaraxastrol	[27]
		21-peroxyhydroxy-taraxastrol acetate	[28]
		30-peroxyhydroxy-pseudotaraxastrol acetate	[28]
		taraxastatin,	[29]
		isodonsesquitin A,	[29]
		taraxastatin B	[29]
		sesquiterpene lactone	[29]
		artecalin,	[30]
		arsanin	[30]
		desacetylmatricarin	[30]
3	Phenolic acids	*p*-hydroxybenzoic acid	[26,28,31,32]
		phenylacetic acid	[26,28,31,32]
		protocatechuic acid	[26,28,31,32]
		p-coumaric acid	[26,28,31,32]
		caffeic acid	[26,28,31,32]
		ferulic acid	[26,28,31,32]
4	Polysaccharides	DLP-I	[21]
		DRP-2b	[22]
		DRP-3a	[22]

**Table 2:** The main signaling pathways responsible for the bioactive components from dandelion for the treatment of cancer.

NO	Signaling Pathways	Cancer Types	Cancer Cells Lines or Animals	Targeting Proteins	Components	Reference
1	Cell cycle arrest and anti-proliferation	Gastric cancer	HGC-27, NCI-N87 MKN-28 gastriccells in mice	Cyclin D1, p21, PCNA, EGFR/AKT1	ψ-taraxasterol taraxasterol	[37] [40]
		Hepatocellular carcinoma	SK-Hep1, HepG2, Hepatocellular carcinoma-bearing mice	Hint1, Cyclin D1, Ki-67	taraxasterol	[38,39]
		Breast cancer	MCF-7	p53	dandelion polysaccharides	[41]
2	Induction of cell apoptosis	Pediatric tumor	SH-SY5Y, Kelly	Mitochondrial membrane potential	dandelion extract	[43]
		Breast cancer	MDA-MB-231	Mitochondrial membrane potential, Caspase-9, Caspase-3, Bcl-2	dandelion root extract	[44]
		Tongue cancer	Tca-8113	Bcl-2, PI3K/Akt	dandelion root extract	[45]
		Cervical cancer and Gastric cancer	HeLa, HGC-27, NCI-N87	Bax, Bcl-2, Caspase-9, PI3K/Akt	taraxasterol	[46]
3	Inhibition of invasion and metastasis	Esophageal squamous cell carcinoma	KYSE30, TE-1 KYSE 450, NEC	zinc finger transcription factor-1, zonula occludens-1, N-cadherin, E-cadherin, PI3K/Akt and Ras/Raf/Erk	flavonoids from the ethanol extract of dandelion extract of dandelion	[49] [55]
		Gastric cancer	BGC823, SGC7901	*MMP-2* mRNA, LncRNA CCAT-1	dandelion extract, dandelion root extract	[8,50]
		Thyroid papillary carcinoma	TPC-1, BCPAP	Wnt/β-catenin, MMP-2 and MMP-9	taraxasterol	[51]
		Bladder cancer	EJ	LncRNA α/β hydrolase domain 11 antisense RNA1	ψ-taraxasterol	[54]
		Breast cancer	MDA-MB-231	STAT3, PD-L1, TNF-α, IL-8, iNOS, IL-10, CD206, Arginase-1, TGF-β	dandelion extract	[56]
4	Induction of autophagy	Breast cancer	MCF-7	mTOR/4EBP1, Beclin1, LC3-II, LC3-I	ψ-taraxasterol	[59]
		Colon cancer	HCT116, SW480	RNF31, p53	taraxasterol	[60]
5	Regulation of cellular metabolism	Lung cancer	H1299, A549 A549	Hexokinase Synthesis of PC and glycerophospholipid metabolism	ψ-taraxastero, dandelion extract	[65] [70]
		Gastric cancer	HGC-27	glyceraldehyde-3-phosphate dehydrogenase	taraxasterol	[66]
		Hepatocellular carcinoma	Huh7, HepG2, mice bearing hepatocellular carcinoma Hepa1-6 and H22	hepcidin, ferroportin	dandelion polysaccharides	[68]
6	Inhibition of tumor angiogenesis	Hepatocellular carcinoma	HepG2	hypoxia-inducible factor-1α, VEGF	dandelion polysaccharides	[72]

## Data Availability

No new data were created or analyzed in this study. Data sharing is not applicable to this article.

## References

[1] Li T., Zhang H., Lian M., He Q., Lv M., Zhai L., Zhou J., Wu K., Yi M. (2025). Global status and attributable risk factors of breast, cervical, ovarian, and uterine cancers from 1990 to 2021. J. Hematol. Oncol..

[2] Filho A.M., Laversanne M., Ferlay J., Colombet M., Piñeros M., Znaor A., Parkin D.M., Soerjomataram I., Bray F. (2025). The GLOBOCAN 2022 cancer estimates: Data sources, methods, and a snapshot of the cancer burden worldwide. Int. J. Cancer.

[3] Ngwa W., Irabor O.C., Schoenfeld J.D., Hesser J., Demaria S., Formenti S.C. (2018). Using immunotherapy to boost the abscopal effect. Nat. Rev. Cancer.

[4] Dutta S., Mahalanobish S., Saha S., Ghosh S., Sil P.C. (2019). Natural products: An upcoming therapeutic approach to cancer. Food Chem. Toxicol..

[5] Ma L., Zhang M., Zhao R., Wang D., Ma Y., Li A. (2021). Plant Natural Products: Promising Resources for Cancer Chemoprevention. Molecules.

[6] Liu Y., Yang S., Wang K., Lu J., Bao X., Wang R., Qiu Y., Wang T., Yu H. (2020). Cellular senescence and cancer: Focusing on traditional Chinese medicine and natural products. Cell Prolif..

[7] Wang S., Hao H.F., Jiao Y.N., Fu J.L., Guo Z.W., Guo Y., Yuan Y., Li P.P., Han S.Y. (2022). Dandelion extract inhibits triple-negative breast cancer cell proliferation by interfering with glycerophospholipids and unsaturated fatty acids metabolism. Front. Pharmacol..

[8] Zhu H., Zhao H., Zhang L., Xu J., Zhu C., Zhao H., Lv G. (2017). Dandelion root extract suppressed gastric cancer cells proliferation and migration through targeting lncRNA-CCAT1. Biomed. Pharmacother..

[9] Yang K., Wang Y. (2024). Dandelion root extracts and taraxasterol inhibit LPS-induced colorectal cancer cell viability by blocking TLR4-NFκB-driven ACE2 and TMPRSS2 pathways. Exp. Ther. Med..

[10] Zhang Y., Hu Y.F., Li W., Xu G.Y., Wang K.R., Li L., Luo H., Zou L., Wu J.S. (2022). Updates and advances on pharmacological properties of Taraxacum mongolicum Hand.-Mazz and its potential applications. Food Chem..

[11] Liu H., Ye J., Yu Y. (2017). Development of taraxacum coarse cereal biscuits. Sci. Technol. Cereals Oils Foods.

[12] Su J., Zhang H., Wang R. (2020). Research on the Processing Technology of Citrus and Dandelion Health Drink. Grain Sci. Technol. Econ..

[13] Sun Y., Wang B., Jiang Q., Yang C., Tang X. (2003). Development and utilization of wild dandelion. Food Res. Dev..

[14] Zhang J., Wen N., Liu Y., Weng R., Tan Z., Chen J., Xu S. (2018). Extraction of Polysaccharide from the Dandelion Root and Development of its Beverage. Farm Prod. Process..

[15] Yarley O.P.N., Kojo A.B., Zhou C., Yu X., Gideon A., Kwadwo H.H., Richard O. (2021). Reviews on mechanisms of in vitro antioxidant, antibacterial and anticancer activities of water-soluble plant polysaccharides. Int. J. Biol. Macromol..

[16] Ni L. (2017). A Kind of Purslane and Dandelion Juice for Removing Rash, as Well as Its Usage, Food, Medicinal Juice, Daily Chemical Product, and Physiotherapy Juice.

[17] Gao Y., Yan L., Ge Q. (2015). A Dandelion Acne-Removing and Beauty-Nourishing Facial Mask and Its Preparation Method.

[18] Shan C. (2010). Research and development of dandelion’s health care value. Heilongjiang Sci. Technol. Inf..

[19] Xue X. (2020). Changes in the Main Active Components and Quality Control During the Processing of Dandelion Black Tea. Master’s Thesis.

[20] Zhao M., Zhou Z.T., Zhang W.D. (2006). Antifugal susceptibility testing and antifugal traditional Chinese medicines screening of oral Candida isolated from head and neck cancer patients treated with radiotherapy or chemotherapy. Hua Xi Kou Qiang Yi Xue Za Zhi.

[21] Wang L., Li T., Liu F., Liu D., Xu Y., Yang Y., Zhao Y., Wei H. (2019). Ultrasonic-assisted enzymatic extraction and characterization of polysaccharides from dandelion (*Taraxacum officinale*) leaves. Int. J. Biol. Macromol..

[22] Cai L., Chen B., Yi F., Zou S. (2019). Optimization of extraction of polysaccharide from dandelion root by response surface methodology: Structural characterization and antioxidant activity. Int. J. Biol. Macromol..

[23] Li S., Shen M. (2013). Content changes of functional components in Taraxacum mongolicum at different harvest times. J. Jilin Agric. Univ..

[24] Hu R., Wang J., Yu H., Jiang S., Liu W., Tang S., Li W. (2023). The Characteristic spectrum-based study on the effect of harvesting time on the chemical composition in taraxacum roots. J. Jilin Agric. Univ..

[25] López-García J., Kuceková Z., Humpolíček P., Mlček J., Sáha P. (2013). Polyphenolic extracts of edible flowers incorporated onto atelocollagen matrices and their effect on cell viability. Molecules.

[26] Xie S., Yang X., Ding Z., Li M., Zhao J. (2011). Chemical Constituents and Pharmacological Effects of Taraxacum mongolicum Hand. Mazz. Nat. Prod. Res. Dev..

[27] Schütz K., Carle R., Schieber A. (2006). Taraxacum--a review on its phytochemical and pharmacological profile. J. Ethnopharmacol..

[28] Berté T.E., Dalmagro A.P., Zimath P.L., Gonçalves A.E., Meyre-Silva C., Bürger C., Weber C.J., Dos Santos D.A., Cechinel-Filho V., de Souza M.M. (2018). Taraxerol as a possible therapeutic agent on memory impairments and Alzheimer’s disease: Effects against scopolamine and streptozotocin-induced cognitive dysfunctions. Steroids.

[29] Zhuang X., Shi W., Shen T., Cheng X., Wan Q., Fan M., Hu D. (2024). Research Updates and Advances on Flavonoids Derived from Dandelion and Their Antioxidant Activities. Antioxidants.

[30] Warashina T., Umehara K., Miyase T. (2012). Constituents from the roots of Taraxacum platycarpum and their effect on proliferation of human skin fibroblasts. Chem. Pharm. Bull..

[31] Shi S., Zhou C., Xu Y., Tao Q., Bai H., Lu F., Lin W., Chen H., Zheng W., Wang L. (2008). Studies on chemical constituents from herbs of Taraxacum mongolicum. China J. Chin. Mater. Medica.

[32] Peng D., Gao J., Guo X., Wang J., Zhang S. (2014). Chemical constituents from roots of Taraxacum mongolicum. Chin. Tradit. Pat. Med..

[33] Huang Y., Wu P., Ying J., Dong Z., Chen X.D. (2021). Mechanistic study on inhibition of porcine pancreatic α-amylase using the flavonoids from dandelion. Food Chem..

[34] Han J.Y., Jo H.J., Kwon E.K., Choi Y.E. (2019). Cloning and Characterization of Oxidosqualene Cyclases Involved in Taraxasterol, Taraxerol and Bauerenol Triterpene Biosynthesis in Taraxacum coreanum. Plant Cell Physiol..

[35] Yan Q., Xing Q., Liu Z., Zou Y., Liu X., Xia H. (2024). The phytochemical and pharmacological profile of dandelion. Biomed. Pharmacother..

[36] Selvaraj C. (2023). Therapeutic targets in cancer treatment: Cell cycle proteins. Adv. Protein Chem. Struct. Biol..

[37] Huo B., Song Y., Tan B., Li J., Zhang J., Zhang F., Chang L. (2022). Research on the mechanisms of taraxerol for the treatment of gastric cancer effect based on network pharmacology. Int. J. Immunopathol. Pharmacol..

[38] Bao T., Ke Y., Wang Y., Wang W., Li Y., Wang Y., Kui X., Zhou Q., Zhou H., Zhang C. (2018). Taraxasterol suppresses the growth of human liver cancer by upregulating Hint1 expression. J. Mol. Med..

[39] Ren F., Zhang Y., Qin Y., Shang J., Wang Y., Wei P., Guo J., Jia H., Zhao T. (2022). Taraxasterol prompted the anti-tumor effect in mice burden hepatocellular carcinoma by regulating T lymphocytes. Cell Death Discov..

[40] Chen W., Li J., Li C., Fan H.N., Zhang J., Zhu J.S. (2020). Network pharmacology-based identification of the antitumor effects of taraxasterol in gastric cancer. Int. J. Immunopathol. Pharmacol..

[41] Niu H. (2017). The Effect of Dandelion Polysaccharides on the Proliferation and Apoptosis in Breast Cancer Cells. Ph.D. Thesis.

[42] Li H., Fan J., Zhao Y., Yang J., Xu H., Manthari R.K., Cheng X., Wang J., Wang J. (2021). Calcium alleviates fluoride-induced kidney damage via FAS/FASL, TNFR/TNF, DR5/TRAIL pathways in rats. Ecotoxicol. Environ. Saf..

[43] Menke K., Schwermer M., Felenda J., Beckmann C., Stintzing F., Schramm A., Zuzak T.J. (2018). *Taraxacum officinale* extract shows antitumor effects on pediatric cancer cells and enhance mistletoe therapy. Complement. Ther. Med..

[44] Mou W., Zhang P., Cui Y., Yang D., Zhao G., Xu H., Zhang D., Liang Y. (2025). Mechanistic Study on the Inhibitory Effect of Dandelion Extract on Breast Cancer Cell Proliferation and Its Induction of Apoptosis. Biology.

[45] Chang H., Zhang D., Guo G., Xiao R. (2020). The aqueous extract of dandelion root regulates apoptosis and migration of human tongue cancer cell line Tca-8113 through the phosphatidylinositol 3-kinase-protein kinase B pathway. Chin. Remedies Clin..

[46] Yao X., Lu B., Lü C., Bai Q., Yan D., Xu H. (2017). Taraxerol Induces Cell Apoptosis through A Mitochondria-Mediated Pathway in HeLa Cells. Cell J..

[47] Liu S.J., Dang H.X., Lim D.A., Feng F.Y., Maher C.A. (2021). Long noncoding RNAs in cancer metastasis. Nat. Rev. Cancer.

[48] Mittal V. (2018). Epithelial Mesenchymal Transition in Tumor Metastasis. Annu. Rev. Pathol..

[49] Sang L., Wu J., Cao Y., Bai X., Bao X. (2020). Effects of Flavonoids Extract from Dandelion on the Proliferation and Invasion of Human Esophageal Squamous Cell Carcinoma. Sci. Technol. Food Ind..

[50] Guo Q. (2015). The Effect of Dandelion on Human Gastric Cancer BGC823 Cells and Mouse Liver Cancer H22 Cells. Master’s Thesis.

[51] Zhu J., Li X., Zhang S., Liu J., Yao X., Zhao Q., Kou B., Han P., Wang X., Bai Y. (2021). Taraxasterol inhibits TGF-β1-induced epithelial-to-mesenchymal transition in papillary thyroid cancer cells through regulating the Wnt/β-catenin signaling. Hum. Exp. Toxicol..

[52] Zhang L., Ge S., Cao B. (2020). Long non-coding RNA MIAT promotes cervical cancer proliferation and migration. J. Biochem..

[53] Han L., Zhou W., Wu F. (2021). Long non-coding RNA LOC284454 promotes hepatocellular carcinoma cell invasion and migration by inhibiting E-cadherin expression. Oncol. Rep..

[54] Meng R., Zheng X., Zhu L., Wang L., Wu L. (2022). Effect of Dandelion Terpenol on Invasion and Metastasis of Bladder Cancer Cells Through Regulating Epithelial Mesenchymal Transition Mediated by ABHD11-AS1. J. Chin. Oncol..

[55] Duan X., Pan L., Deng Y., Liu Y., Han X., Fu H., Li Y., Li M., Wang T. (2021). Dandelion root extract affects ESCC progression via regulating multiple signal pathways. Food Funct..

[56] Deng X.X., Jiao Y.N., Hao H.F., Xue D., Bai C.C., Han S.Y. (2021). Taraxacum mongolicum extract inhibited malignant phenotype of triple-negative breast cancer cells in tumor-associated macrophages microenvironment through suppressing IL-10/STAT3/PD-L1 signaling pathways. J. Ethnopharmacol..

[57] Liu S., Yao S., Yang H., Liu S., Wang Y. (2023). Autophagy: Regulator of cell death. Cell Death Dis..

[58] Debnath J., Gammoh N., Ryan K.M. (2023). Autophagy and autophagy-related pathways in cancer. Nat. Rev. Mol. Cell Biol..

[59] Zhu K., Ding M., Li Y., Piao Y., Chen L. (2019). Effect of Taraxerol in Inducing Autophagy in Breast Cancer Cells via mTOR Signaling Pathway. Chin. J. Exp. Tradit. Med. Formulae.

[60] Tang C.T., Yang J., Liu Z.D., Chen Y., Zeng C. (2021). Taraxasterol acetate targets RNF31 to inhibit RNF31/p53 axis-driven cell proliferation in colorectal cancer. Cell Death Discov..

[61] Mao Y., Xia Z., Xia W., Jiang P. (2024). Metabolic reprogramming, sensing, and cancer therapy. Cell Rep..

[62] Yang K., Wang X., Song C., He Z., Wang R., Xu Y., Jiang G., Wan Y., Mei J., Mao W. (2023). The role of lipid metabolic reprogramming in tumor microenvironment. Theranostics.

[63] Tan Y., Li J., Zhao G., Huang K.C., Cardenas H., Wang Y., Matei D., Cheng J.X. (2022). Metabolic reprogramming from glycolysis to fatty acid uptake and beta-oxidation in platinum-resistant cancer cells. Nat. Commun..

[64] Li Z., Zhang H. (2016). Reprogramming of glucose, fatty acid and amino acid metabolism for cancer progression. Cell Mol. Life Sci..

[65] Liu D., Zhao P., Wang L., Lan F., Wei X., Zhang H. (2018). Effect of taraxerol on proliferation and glycolysis of lung cancer cells. J. Clin. Pathol. Res..

[66] Zhao Y., Zhang L., Guo M., Yang H. (2021). Taraxasterol suppresses cell proliferation and boosts cell apoptosis via inhibiting GPD2-mediated glycolysis in gastric cancer. Cytotechnology.

[67] Chen Y., Fan Z., Yang Y., Gu C. (2019). Iron metabolism and its contribution to cancer (Review). Int. J. Oncol..

[68] Ren F., Yang Y., Wu K., Zhao T., Shi Y., Song M., Li J. (2021). The Effects of Dandelion Polysaccharides on Iron Metabolism by Regulating Hepcidin via JAK/STAT Signaling Pathway. Oxid. Med. Cell Longev..

[69] Bian X., Liu R., Meng Y., Xing D., Xu D., Lu Z. (2021). Lipid metabolism and cancer. J. Exp. Med..

[70] Man J., Wu L., Han P., Hao Y., Li J., Gao Z., Wang J., Yang W., Tian Y. (2022). Revealing the metabolic mechanism of dandelion extract against A549 cells using UPLC-QTOF MS. Biomed. Chromatogr..

[71] Liu Z.L., Chen H.H., Zheng L.L., Sun L.P., Shi L. (2023). Angiogenic signaling pathways and anti-angiogenic therapy for cancer. Signal Transduct. Target. Ther..

[72] Ren F., Wu K., Yang Y., Yang Y., Wang Y., Li J. (2020). Dandelion Polysaccharide Exerts Anti-Angiogenesis Effect on Hepatocellular Carcinoma by Regulating VEGF/HIF-1α Expression. Front. Pharmacol..

[73] Rezaie H., Alipanah-Moghadam R., Jeddi F., Clark C.C.T., Aghamohammadi V., Nemati A. (2023). Combined dandelion extract and all-trans retinoic acid induces cytotoxicity in human breast cancer cells. Sci. Rep..

[74] Qu J., Ke F., Yang X., Wang Y., Xu H., Li Q., Bi K. (2022). Induction of P-glycoprotein expression by dandelion in tumor and heart tissues: Impact on the anti-tumor activity and cardiotoxicity of doxorubicin. Phytomedicine.

[75] Nguyen C., Mehaidli A., Baskaran K., Grewal S., Pupulin A., Ruvinov I., Scaria B., Parashar K., Vegh C., Pandey S. (2019). Dandelion Root and Lemongrass Extracts Induce Apoptosis, Enhance Chemotherapeutic Efficacy, and Reduce Tumour Xenograft Growth In Vivo in Prostate Cancer. Evid. Based Complement. Altern. Med..

[76] Kang L., Miao M.S., Song Y.G., Fang X.Y., Zhang J., Zhang Y.N., Miao J.X. (2021). Total flavonoids of Taraxacum mongolicum inhibit non-small cell lung cancer by regulating immune function. J. Ethnopharmacol..

[77] Li Y., Deng Y., Zhang X., Fu H., Han X., Guo W., Zhao W., Zhao X., Yu C., Li H. (2022). Dandelion Seed Extract Affects Tumor Progression and Enhances the Sensitivity of Cisplatin in Esophageal Squamous Cell Carcinoma. Front. Pharmacol..

[78] Qu J., Ke F., Liu Z., Yang X., Li X., Xu H., Li Q., Bi K. (2022). Uncovering the mechanisms of dandelion against triple-negative breast cancer using a combined network pharmacology, molecular pharmacology and metabolomics approach. Phytomedicine.

